# Single-cell transcriptomics reveal PD-1-loss-driven immune dysregulation of pulmonary lymphocytes during early *Mycobacterium tuberculosis* infection

**DOI:** 10.1128/spectrum.01199-26

**Published:** 2026-07-24

**Authors:** Bing Yang, Haoqi Wang, Siqi Zhang, Jinru Cai, Gang Cao, Xuan Ma, Jinxia Dai

**Affiliations:** 1 State Key Laboratory of Agricultural Microbiology, Huazhong Agricultural University 124443, Wuhan, China; 2 College of Veterinary Medicine, Huazhong Agricultural University 627716, Wuhan, China; 3 College of Life Sciences and Technology, Huazhong Agricultural University 627727, Wuhan, China; 4 Faculty of Life and Health Sciences, Shenzhen University of Advanced Technology, Chinese Academy of Sciences 644974, Shenzhen, China; 5 College of Agriculture, Ningxia University 56693, Yinchuan, China; Children's National Hospital, George Washington University, Washington, DC, USA

**Keywords:** programmed cell death receptor 1, *Mycobacterium tuberculosis*, pulmonary lymphocytes, transcriptomic landscape, transcriptional regulatory networks, cellular communication

## Abstract

Although PD-1 blockade shows strong antitumor efficacy and is being considered for chronic infections including *Mycobacterium tuberculosis* (*M.tb*), its influence on early lymphocyte responses to *M.tb* remains poorly understood. In this study, we characterized the transcriptional and developmental states of pulmonary B and T cells in mice lacking PD-1 after 7 days post-*M.tb* infection. We found that loss of PD-1 disturbed pulmonary lymphocyte homeostasis, characterized by abnormal regulatory T cell (Tregs) expansion, early exhaustion-like differentiation of cytotoxic T cells, and impaired maturation of memory B cells. These alterations are associated with changes in key transcriptional regulators, including reduced activity of factors promoting effector differentiation (e.g., *Runx2*, *Runx3* in T cells; *Tcf4*, *Pou2f2* in B cells) and upregulation of regulators driving regulatory or suppressive phenotypes (e.g., *Ikzf2* in Tregs). Additionally, PD-1 deficiency rewired B-T cell communication with diminished antigen presentation and co-stimulation while amplifying proinflammatory signals. These insights provide a transcriptional blueprint for PD-1-mediated immune balance, with implications for host-directed therapies in *tuberculosis*.

**IMPORTANCE:**

While PD-1 inhibition can boost protective T cell responses, it has also been linked to increased TB susceptibility. Our study reveals that PD-1 plays a previously unrecognized role in shaping the earliest immune responses to *M.tb*. In mice lacking PD-1, lung lymphocytes failed to develop in a balanced manner: regulatory T cells expanded excessively, cytotoxic T cells showed signs of early exhaustion, and memory B cell maturation was delayed. These changes disrupted the normal communication between B and T cells, weakening adaptive immunity while amplifying inflammation. By defining PD-1 as a critical organizer of early lymphocyte fate, our findings highlight the risks of indiscriminate PD-1 blockade in infectious contexts and point toward more precise strategies for host-directed TB therapies and vaccine design.

## INTRODUCTION

*Tuberculosis* (TB), a contagious infectious disease caused by *Mycobacterium tuberculosis* (*M.tb*), remains a formidable global health challenge, with an estimated 10.70 million new active cases and 1.20 million deaths in 2024 (1). Despite substantial advances in TB prevention, diagnosis, and treatment, timely and accurate diagnosis remains a major challenge, particularly in resource-limited settings with a high disease burden. Recent developments in molecular diagnostic technologies, including nucleic acid amplification tests (NAATs), have significantly improved TB detection (2). Among these, the TB loop-mediated isothermal amplification (TB-LAMP) assay has been included in the World Health Organization (WHO) List of Essential *In Vitro* Diagnostics and is recommended by WHO for the diagnosis of active pulmonary TB in primary healthcare settings (3, 4). Nevertheless, TB continues to impose a considerable public health burden worldwide, highlighting the need for a deeper understanding of host-pathogen interactions and immune regulatory mechanisms involved in disease progression. Murine models of *M.tb* infection have proven indispensable for elucidating host-pathogen interactions, providing valuable insights into the immune mechanisms underlying tuberculosis, despite species-specific differences in granuloma formation (5). While innate immunity initiates early defense mechanisms against *M.tb* through phagocytic cells and inflammatory mediators (6, 7); however, these responses alone fail to control bacterial proliferation. Ultimately, pathogen clearance relies on the coordinated actions of adaptive immunity, particularly the orchestration of T and B lymphocyte responses (8–11). Despite advances, the transcriptional programs regulating T and B lymphocyte behavior during early *M.tb* exposure remain poorly characterized.

As central mediators of adaptive immunity, T and B cells employ multifaceted mechanisms to combat *M.tb*. CD4^+^ helper T cells exert their protective role through cytokine secretion (e.g., IL-2, IFN-γ, and TNF-α) and direct cellular interactions, with their depletion correlating with enhanced bacterial dissemination (12, 13). CD8^+^ cytotoxic T lymphocytes (CTLs) contribute by eliminating infected cells via perforin/granzyme release and Fas-FasL signaling (14, 15). B cells participate through antibody production, antigen presentation via MHC-II, and modulation of T-cell responses (16–18). Crucially, inter-lymphocyte communication, mediated by cytokine networks and receptor-ligand interactions, finely balances protective immunity with the risk of immunopathology. For example, CD4^+^ T cell help sustains CTL functionality and prevents exhaustion during murine TB (19), while effector and regulatory B cells influence T-cell priming via antigen presentation (20). Additionally, follicular helper T (Tfh) cells promote B-cell differentiation through CD40L-CD40 and ICOSL-ICOS interactions coupled with IL-21 secretion (21–23). These intricate interdependencies underscore the necessity of spatial and functional coordination among lymphocyte subsets for effective anti-mycobacterial immunity.

While successful control of *M.tb* infection, host immune system should avoid excessive inflammation by multifaceted regulations. In this case, the inhibitory immune checkpoint receptors, such as programmed cell death-1 (PD-1), play its immunosuppressive effects to adjust the host immune response and maintain self-tolerance. PD-1 expression is significantly upregulated during various infectious diseases, including *M.tb* (24), hepatitis B, hepatitis C, and human immunodeficiency virus (HIV) infection, where it often negatively modulates pathogen clearance. In that way, PD-1 may be involved in the regulation of anti-infective responses, suggesting that the anti-PD-1 therapy may have potential applications in the prevention and treatment of infectious diseases, including TB. Paradoxically, clinical reports have documented TB re-activation in patients undergoing anti-PD-1 immunotherapy for cancer, raising concerns about the complex role of PD-1 in infection control (25–27). Several studies suggested that PD-1 blockade exacerbates *M.tb* infection, primarily during the adaptive immunity phase. PD-1-deficient mice exhibit heightened susceptibility to *M.tb*, developing severe necrotic pneumonia and drastically reduced survival at 8 weeks post-infection (28). Although the underlying mechanisms remain incompletely defined, excessive IFN-γ production by adaptive immune cells, particularly CD4^+^ T cells, contributes substantially to lethal immunopathology in these mice (29, 30). Rhesus macaques treated with 13-14 weeks of anti-PD-1 monoclonal antibody developed higher levels of inflammation and increased granuloma bacterial loads than isotype control-treated animals, implicating caspase 1 activation in disease exacerbation following PD-1 blockade (31). Given the chronic progression of TB, the early immune events following infection usually influenced long-term immune fates and disease trajectories. While prior researches have focused on innate immune cell populations during the initial infection stages, the impact of PD-1 ablation on lymphocyte responses at single-cell resolution during early *M.tb* infection remains unexplored.

In this study, we employed single-cell RNA sequencing to delineate the early immunological consequences of PD-1 deficiency in *M.tb*-infected mice. We reveal that PD-1 loss induces profound transcriptional and developmental perturbations across pulmonary lymphocyte subsets, characterized by expanded Treg activation, cytotoxic T cell exhaustion, and impaired memory B cell differentiation. These aberrations coincide with dysregulated expression of lineage-defining transcription factors and disrupted B-T cell communication networks, collectively undermining the establishment of adaptive immunity. Our findings illuminate the dual role of PD-1 as both an immune checkpoint and a critical coordinator of early anti-mycobacterial responses.

## MATERIALS AND METHODS

### Mice

Wild-type female BALB/c mice were obtained from the Experimental Animal Center of Huazhong Agricultural University. PD-1 deficient (PD-1^−/−^) female BALB/c mice were kindly provided by Prof. Min Cui (Huazhong Agricultural University). Genotyping of PD-1 knockout mice was performed by PCR using the following primers:

PDwt5: 5′-GCCAGCTAAGAGGCCACAGCTA-3′;

PD970R: 5′-CGGTGCTCTCTGTGGAGGGTCTG-3′; mPD-1KO31: 5′-TTGTGTAGCGCCAAGTGCCCAGCG-3′.

All mice were maintained under specific pathogen-free (SPF) conditions, with a standard 12-h light-dark cycle, controlled temperature of 20°C–25°C and relative humidity of 40%–50%. Water and food were provided and libitum. Experiments were run with 8- to 10-week-old mice.

### *M.tb* strain and mouse infection

*M.tb* strain H37Ra used in this study was obtained from the American Type Culture Collection (ATCC25177) and was cultured at 37°C in Middlebrook 7H9 broth (BD Biosciences, Cat. No. 271310) supplemented with 0.5% glycerin (Sigma Aldrich, Cat. No. G9012), 0.05% Tween 80 (Sigma Aldrich, Cat. No. P1754) and 10% Middlebrook Oleic Albumin Dextrose Catalase (OADC) (BD Biosciences, Cat. No. 212240) until mid-logarithmic phase (OD₆₀₀ ≈ 0.6–0.8). Aliquots of this culture were stored at −80°C in 15% glycerol as frozen stocks.

For infection experiments, an aliquot was thawed, diluted 1:10 in fresh 7H9 medium, and allowed to recover for 4 h at 37°C with gentle shaking before use. The bacterial concentration of the inoculum was adjusted to the desired dose (1.0 × 10⁶ CFU per 100 µL) based on OD₆₀₀ readings and further verified by plating serial 10-fold dilutions on Middlebrook 7H11 agar (BD Biosciences, Cat. No. 283810) supplemented with 0.5% glycerin and 10% OADC. Infection of BALB/c mice was performed with 1.0 × 10^6^ colony-forming units (CFUs) of *M.tb* H37Ra suspended in 100 μL of sterile PBS by the intravenous route. Infected mice were humanely euthanized on day 7 post-infection for subsequent analyses.

Both bacterial growth and infection experiments were performed in biosafety level 2 (BLS-2) facilities of Huazhong Agricultural University (Wuhan, China).

### Tissue dissociation and single-cell suspension preparation

For lung tissue, freshly harvested lungs from mice at day 7 post‐*M.tb* infection were dissected into 2–3 mm^3^ fragments and enzymatically digested in RPMI 1640 medium supplemented with 10% fetal bovine serum (FBS), 40 IU/mL DNase I (Vazyme, Cat. No. EN401) and 1 mg/mL type I collagenase from *Clostridium histolyticum* (ThermoFisher, Cat. No.17100017) at 37°C for 60 min with gentle agitation. The digested tissues were passed through a 70-μm cell strainer to obtain a single-cell suspension, followed by centrifugation at 1,500 rpm for 5 min. Red blood cells were lysed using red blood lysis buffer at 4°C for 5 min. The remaining cell pellets were washed three times with RPMI-1640 and resuspended in PBS supplemented with 0.2% bovine serum albumin (BSA). The resulting single-cell suspensions were subsequently used for single-cell RNA sequencing and flow cytometry according to the procedures described below.

For spleens and lung-draining mediastinal lymph node, tissues were mechanically dissociated by gentle grinding through sterile 40-μm cell strainer. Cell suspensions were collected using RPMI-1640 medium and centrifuged at 1,500 rpm for 5 min. Red blood cells were lysed as described above, and remaining cells were washed in RPMI-1640 medium three times. Final cell pellets were resuspended in PBS supplemented with 0.2% BSA for subsequent experiments.

### Flow cytometry

Prepared single-cell suspensions from individual samples were washed three times with PBS supplemented with 0.2% BSA. Then, cells were stained with fluorescence-conjugated antibodies in 100-μL flow cytometry staining buffer (BD Biosciences, Cat. No. 563794) for 30 min at 4°C in the dark. For the immunophenotyping of B and T lymphoid cells, the prepared cells were stained with anti-CD45-BV750 (Biolegend, Cat. No. 103157), anti-CD3e-PE (Biolegend, Cat. No. 100205), anti-CD19b-APC (Biolegend, Cat. No. 152410). T cells were gated as CD45^+^CD3^+^ cells, and B cells were gated as CD45^+^CD3^-^CD19^+^ cells.

To phenotype the subsets of B and T cells, the prepared cells were stained with anti-CD45-BV750 (Biolegend, Cat. No. 103157), anti-CD3e-Pacific Blue (Biolegend, Cat. No. 100205), anti-CD4-FITC (Biolegend, Cat. No. 100405), anti-CD8a-APC/Cy7 (Biolegend, Cat. No. 155015), anti-CD44-PerCP/Cy5.5 (Biolegend, Cat. No. 103031), anti-CD62L-PE/Cy7 (Biolegend, Cat. No. 104418), anti-CD19b-APC (Biolegend, Cat. No. 152410), and anti-CD27-PE (Biolegend, Cat. No. 124209). Naive T cells were gated on CD44^-^CD62L^+^ cells from CD4^+^ and CD8^+^ T cell populations. Effector T cells were gated on CD44^−^CD62L^−^ cells in CD3^+^ T cell populations. Memory B cells were gated on CD3^-^CD19^+^CD27^+^ cells.

To phenotype cytotoxic T cells, freshly prepared cells were surface-stained with anti-CD45-BV750 (Biolegend, Cat. No. 103157), anti-CD3e-Pacific Blue (Biolegend, Cat. No. 100205), and anti-CD8-FITC (Biolegend, Cat. No. 100706) for 1 h at 4°C in the dark. Cells were then fixed and permeabilized with Cytofix/Cytoperm Fixation/Permeabilization Kit (BD Biosciences, Cat. No. 554714) for 20 min at 4°C and stained intracellularly by overnight incubation at 4°C in the dark with anti-Granzyme B-PE (Biolegend, Cat. No. 372207) in 1× BD Perm/Wash buffer. Cytotoxic T cells were gated on CD8^+^Granzyme B^+^ populations.

To phenotype Tregs, freshly prepared cells were surface-stained with anti-CD45-BV750 (Biolegend, Cat. No. 103157), anti-CD3e-Pacific Blue (Biolegend, Cat. No. 100205), anti-CD4-FITC (Biolegend, Cat. No. 100405), and anti-CD25-APC (Biolegend, Cat. No. 113708) for 1 h at 4°C in the dark. Cells were then fixed and permeabilized with eBioscience Foxp3/Transcription Factor Staining Buffer Set (Invitrogen, Cat. No. 00-5523) and stained intracellularly by overnight incubation at 4°C in the dark with anti-FOXP3-PE (Biolegend, Cat. No. 118903) in 1× Permeabilization Buffer (Invitrogen, Cat. No. 00-8333). Tregs were gated on CD25^+^FOXP3^+^ cells from CD4^+^ T cell populations.

All samples were acquired by cytoFLEX-LX flow cytometer (Beckman Coulter) and analyzed with CytExpert software (Beckman Coulter) or FlowJo software.

### Single-cell RNA sequencing (scRNA-seq)

For single-cell transcriptomic analysis, total lung single-cell suspensions were prepared from each experimental group without prior enrichment for specific immune cell populations. Briefly, lungs from three mice per group were pooled and enzymatically digested to obtain a single-cell suspension. Cell viability was assessed using propidium iodide (PI) (ThermoFisher, Cat. No. P1304MP) and Calcein-AM (Invitrogen, Cat. No. C1430) staining, according to the manufacturer’s instructions. Only samples with cell viability greater than 80% were used for downstream processing.

High-throughput scRNA-seq was performed using MobiDrop single-cell sequencing kit. Briefly, the single-cell suspension derived from dissociated lung tissues of mice was analyzed for viability, and then loaded into the microfluidic chip of ChipA Single Cell Kit v2.0 (MobiDrop (Zhejiang) Co., Ltd., Cat. No. S050100201) to generate droplets using MobiNova-100 (MobiDrop (Zhejiang) Co., Ltd., Cat. No. A1A40001). Each cell was wrapped into a droplet which contained amplification reagent and a gel bead linked with up to millions of oligos (cell unique barcode). After encapsulation, droplets suffer light cut by MobiNovaSP-100 (MobiDrop (Zhejiang) Co., Ltd., Cat. No. A2A40001) following oligos diffusion into amplification mix. The mRNAs were captured by gel beads containing oligo(dT) in droplets. Following reverse transcription, cDNAs with barcodes were amplified, and a library was constructed using the High Throughput Single Cell 3’RNA-Seq Kit v2.0 (MobiDrop (Zhejiang) Co., Ltd., Cat. No. S050200201) and the 3′ Single Index Kit (MobiDrop (Zhejiang) Co., Ltd., Cat. No. S050300201). The scRNA libraries were sequenced on an Illumina NovaSeq 6000 sequencing system (paired-end multiplexing run, 150 bp) by LC-Bio Technology Co., Ltd. (Hangzhou, China).

### The scRNA-seq data analysis and visualization

Raw reads of scRNA-seq were pre-processed to obtain clean reads for further analysis. MobiVision software pipeline was used for clean data to perform quality control, extract barcodes and UMI, and align to the mouse reference genome (GRCm38-PhiX-gencodevM19). The output matrices with the information of cell index and gene expression level were imported into Seurat (version 4.3.0) for downstream analysis. All further analyses were performed using R statistics software version 4.3.0. For data filtering, cells expressing >5% mitochondrial transcripts or fewer than 250 total transcripts were removed as low-quality cells. Normalization of the scRNA-seq data was performed using the SCTransform function of Seurat. The SelectIntegrationFeature function was employed to identify 2,000 highly variable genes for data set integration, followed by principal component analysis (PCA) of individual samples using the RunPCA function. For analyses focused on lymphocyte populations, clusters preliminarily annotated as T cells and B cells were extracted from the integrated full-lung data set based on lymphocyte clusters after preliminary cell annotation, and all downstream sub-clustering and functional analyses were performed specifically on this lymphocyte subset.

Cell clustering was conducted with FindNeighbors and FindCluster functions based on the first 20 principal components. Dimension reduction analysis was performed using the RunUMAP or RunTSNE functions. Cell types were annotated based on cell-type specific marker genes which were characterized with FindAllMarkers function (logFC.threshold = 0.25). Differential expression genes (DEGs) were identified using FindMarkers function (Bonferroni-adjusted *P* < 0.05, |log2[Fold-Change]| > 0.5). Pathway enrichment analyses were performed on these DEGs using the function of ClusterProfiler, and gene identifiers were mapped by using R package “org.Mm.eg.db.” The gene sets used for pathway enrichment were downloaded from GSEA database (https://www.gsea-msigdb.org/gsea), and the gene set scores were calculated using the AddModuleScore function in Seurat. Statistical analysis of the gene set scores between WT and PD-1^−/−^ groups was performed using the Wilcoxon test implemented in the ggpubr package (v.0.6.0) (32). The DEGs and gene sets were visualized as bar plots using the “ggplot2” package.

The cell lineage trajectory of B and T cells was inferred by using Monocle2. We obtained the top 500 DEGs from each subcluster using the differentialGeneTest function, and genes with a *q*-value < 0.05 were used for ordering the cells in pseudotime analysis. Following trajectory construction, the differentialGeneTest function was used to detect genes differentially expressed along pseudotime. The gene regulatory networks (GRNs) were constructed using SCENIC pipeline (v.1.3.1) from single-cell transcriptomic data. Regulon-specific scores (RSSs) were calculated to quantify the cell-type specificity of a regulon. Regulon modules were identified based on the Connection Specificity Index (CSI). For each regulon module, the activity score for a given cell type was calculated as the mean activity score of its regulon members across all cells of that cell type. The top-ranked cell types for each module were subsequently identified. The intercellular communications were inferred using CellChat (v.1.6.1) via the expression of known receptor-ligand pairs.

### Quantification and statistical analysis

Flow cytometric data were acquired using CytExpert software (Beckman Coulter) or FlowJo software, and statistical analyses were performed using GraphPad Prism 10.4.0. Statistical significance was assessed using the unpaired Student’s *t*-test.

Statistical analyses of scRNA-seq data were performed in R unless otherwise specified. Differences between two independent groups were assessed using two-tailed Mann-Whitney U (Wilcoxon rank-sum) tests.

## RESULTS

### PD-1 deficiency alters pulmonary lymphocyte composition during early *M.tb* infection

**Fig 1:**
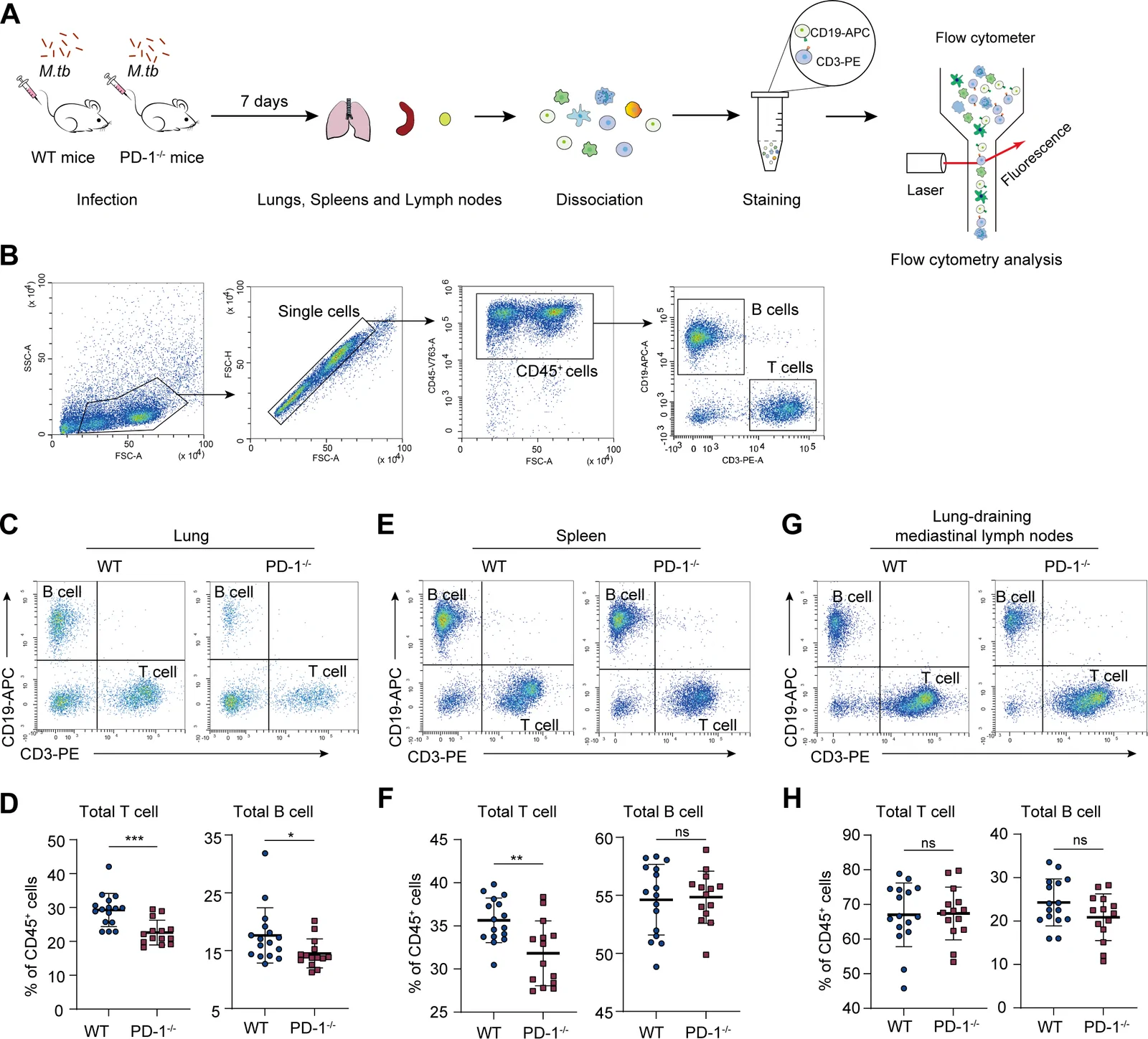
PD-1 deficiency alters lymphocyte composition across multiple organs during early *M.tb* infection. (**A**) Schematic overview of experimental design. (**B**) Flow cytometric gating strategy for the identification of T and B lymphocyte populations. (**C–H**) Representative flow cytometric plots and quantification of total CD3^+^ T cells and CD19^+^ B cells in the lungs (**C, D**), spleens (**E, F**), and lung-draining mediastinal lymph nodes (**G, H**) from WT and PD-1^−/−^ mice at 7 days post-*M.tb* infection. Data are pooled from two independent experiments, with *n* = 16 mice in WT group and *n* = 14 mice in the PD-1^−/−^ group. Data are represented as mean ± SD. Statistical significance was assessed using the unpaired Student’s *t*-test. *P < 0.05; **P < 0.01; ***P < 0.001; ns, not significant.

To investigate PD-1’s role in regulating early immune responses to *M.tb*, we quantified B and T lymphocyte frequencies in lungs, spleens, and lung-draining mediastinal lymph nodes (medLNs) of WT and PD-1^−/−^ mice at 7 days post-infection (dpi) using flow cytometry (Fig. 1A and B). Compared with WT controls, PD-1^−/−^ mice exhibited significantly reduced pulmonary B and T cell frequencies (Fig. 1C and D). A similar decrease was observed for splenic T cells, although splenic B cell proportions remained unchanged (Fig. 1E and F). In contrast, no significant differences in B or T cell frequencies were observed in medLNs (Fig. 1G and H), suggesting PD-1’s regulatory effects on lymphocyte responses are tissue-specific during the early phase of *M.tb* infection.

**Fig 2:**
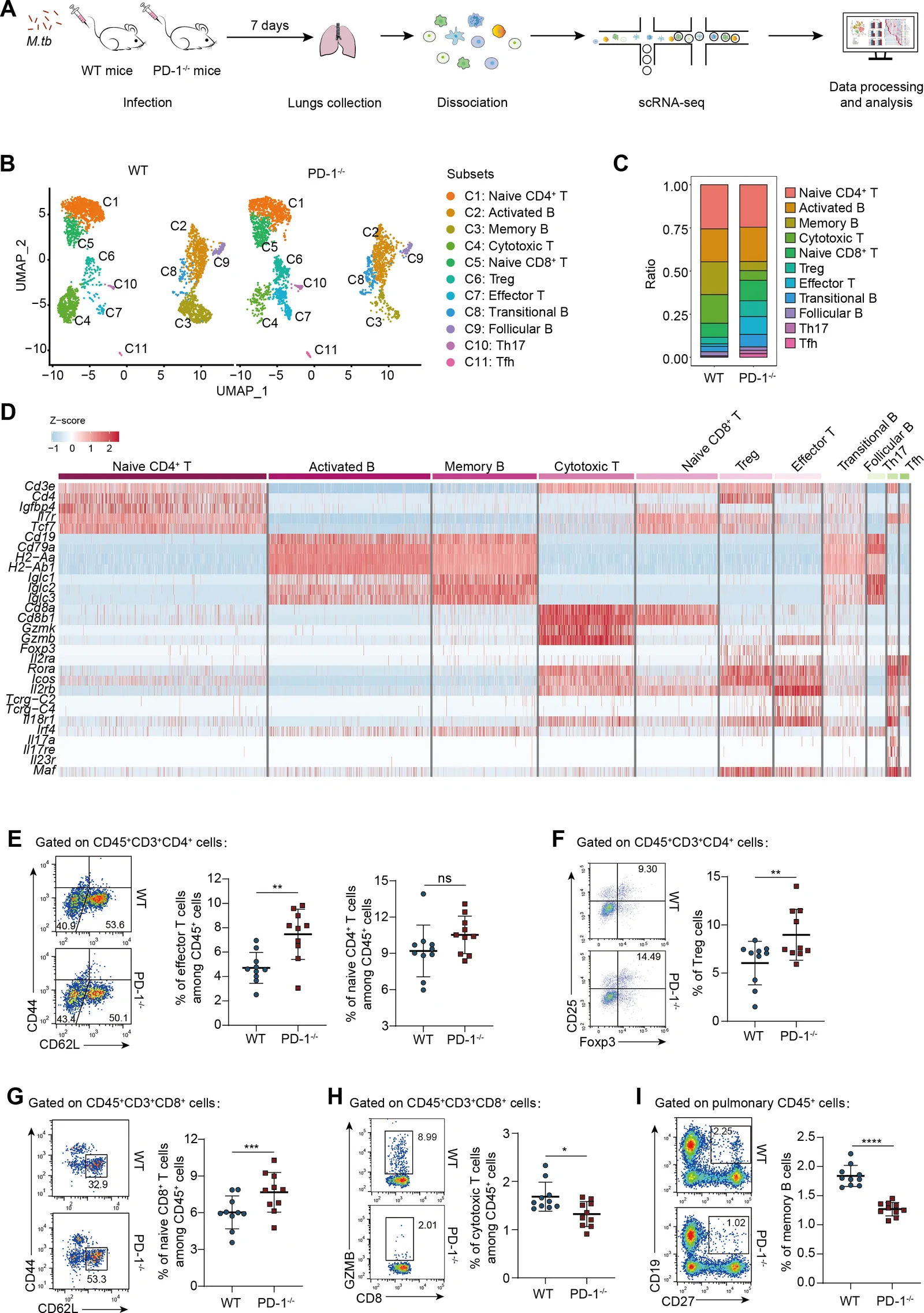
PD-1 deficiency reshapes pulmonary lymphocyte composition during early *M.tb* infection. (**A**) Schematic representation of the single-cell RNA sequencing workflow performed on lung tissues from WT and PD-1^−/−^ mice at day 7 post-*M.tb* infection. (**B**) Uniform manifold approximation and projection (UMAP) visualization of integrated single-cell transcriptomic profiles, with each cluster color-coded by annotated B and T cell types from *M.tb*-infected WT and PD-1^−/−^ mice. (**C**) Comparative proportions of identified lymphocyte subpopulations in the lungs of *M.tb*-infected WT and PD-1^−/−^ mice. (**D**) Heatmap showing the expression patterns of canonical marker genes for cluster annotation. (**E–I**) Representative flow cytometric plots and quantification of specific lymphocyte subsets in the lungs of WT and PD-1^−/−^ mice at 7 days post-infection, including effector and naive CD4^+^ T cells (**E**), Tregs (**F**), naive CD8^+^ T cells (**G**), cytotoxic T cells (**H**), and memory B cells (**I**). Data are pooled from two independent experiments, with *n* = 10 mice per group for flow cytometry analysis. Data are represented as mean ± SD. Statistical significance was assessed using the unpaired Student’s *t*-test. *P < 0.05; **P < 0.01; ***P < 0.001; ****P < 0.0001; ns, not significant.

To further characterize the transcriptional heterogeneity of pulmonary B and T cells between WT and PD-1^−/−^ mice, we performed scRNA-seq on pulmonary immune cells at 7 dpi (Fig. 2A). Unsupervised clustering identified four major B cell subsets (activated B cells, memory B cells, transitional B cells, and follicular B cells) and seven T cell subsets (naive CD4^+^ T cells, cytotoxic T cells, naive CD8^+^ T cells, Tregs, effector T cells, Th17 cells, and Tfh cells) based on the expression of canonical lineage-specific markers (Fig. 2B and D).

Comparative analysis of cell subset proportions revealed that genetic PD-1 ablation skewed pulmonary lymphocyte composition. PD-1^−/−^ lungs showed increased frequencies of Tregs, effector T, Th17, and Tfh cells, alongside a notable reduction in cytotoxic T cells (Fig. 2C). Similarly, transitional B cells were expanded in PD-1^−/−^ mice lungs, accompanied by a reduction in memory B cells (Fig. 2C). These compositional shifts were further validated by flow cytometry analysis (Fig. S1A through C). Specifically, compared with WT controls, PD-1^−/−^ lungs exhibited a marked increase in effector T cells (Fig. 2E), Tregs (Fig. 2F), and naive CD8^+^ T cells (Fig. 2G), while cytotoxic T cells (Fig. 2H) and memory B cells (Fig. 2I) were significantly reduced. Together, these data indicate that PD-1 plays a critical role in orchestrating the early differentiation and subset distribution of pulmonary lymphocytes during *M.tb* infection.

### Transcriptional reprogramming of pulmonary T lymphocytes under PD-1 deficiency during early *M.tb* infection

**Fig 3:**
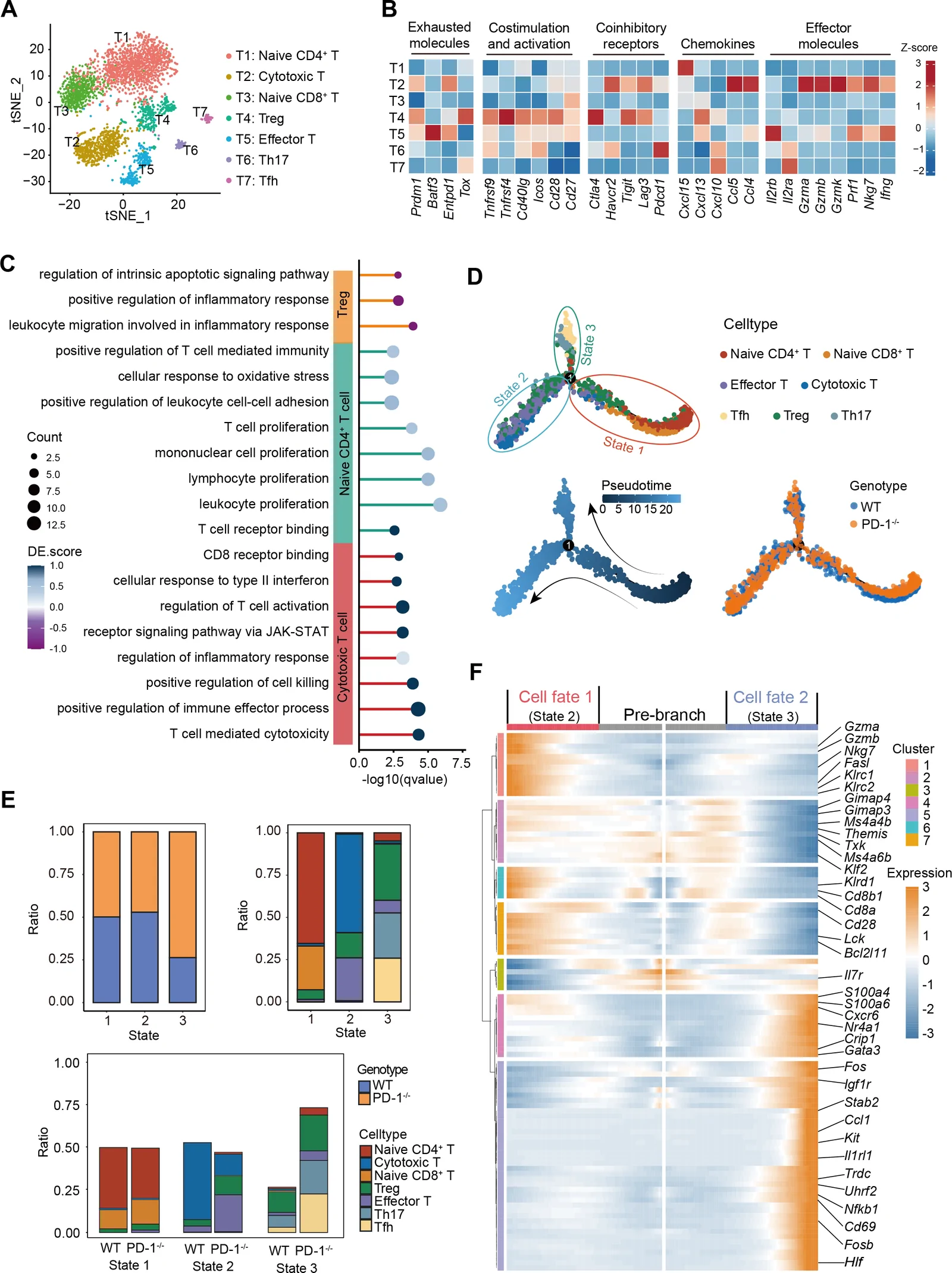
PD-1 deficiency reprograms the transcriptomic landscape and differentiation trajectory of pulmonary T lymphocytes during early *M.tb* infection. T cell subsets were extracted from pooled lung cells of three mice per group. (**A**) *t*-distributed stochastic neighbor embedding (t-SNE) plot of lung-derived T cells from WT and PD-1^−/−^ mice, with color-coded by annotated T cell subsets. (**B**) Heatmap of scaled and normalized expression levels for representative genes across T cell subclusters. (**C**) Enriched pathways of differentially expressed genes across T cell subsets between WT and PD-1^−/−^ groups. The color scale represents the absolute value of differential expression (DE) score. Positive values indicate that >50% of genes in the pathway are upregulated, while negative values indicate predominant downregulation. (**D**) Pseudotime trajectory analysis of T cell differentiation constructed by Monocle. Top: Cell distribution along developmental trajectories annotated by T cell subtype and inferred cell states. T cell subsets and states are labeled by color. bottom left: Cells ordered by pseudotime. Bottom right: Cells grouped by sample origin (WT and PD-1^−/−^). (**E**) Proportional distribution of WT and PD-1^−/−^ cells, T subsets, T subsets from different groups in different states. (**F**) Hierarchical clustering heatmap of differently expressed genes between WT and PD-1^−/−^ T cells along with the pseudotime trajectory. The color scale indicates relative gene expression levels.

The skewed composition of pulmonary T lymphocytes under PD-1 deletion (Fig. 2C, E through I) suggests that PD-1 plays a pivotal role in constraining T cell differentiation toward immunomodulatory lineages during early *M.tb* infection. To uncover the molecular drivers of these cell compositional shifts, we re-clustered pulmonary T cell subsets using the *t*-distributed stochastic neighbor embedding (t-SNE) algorithm and examined their transcriptional profiles (Fig. 3A). Among the identified T cell subsets, cytotoxic T cells in PD-1^−/−^ lungs displayed a dual transcriptional phenotype. These cells simultaneously expressed elevated levels of effector molecules, including *Gzma*, *Gzmb*, *Gzmk*, *Prf*, *Nkg7*, *Ifng*, *Ccl4*, and *Ccl5*, alongside exhaustion-associated genes, such as *Prdm1*, *Entpd1*, *Havcr2*, *Tigit*, and *Lag3* (Fig. 3B). These paradoxical signatures suggest that while PD-1 deficiency triggers premature cytotoxic activation, it fails to sustain long-term functional integrity. In contrast, Tregs in PD-1^−/−^ lungs exhibited signs of hyperactivation, evidenced by upregulated co-stimulatory molecules, including *Tnfrsf9*, *Tnfrsf4*, *Cd40lg*, *Icos*, *Cd28*, and *Cd27* (Fig. 3B), indicating enhanced suppressive potential. Meanwhile, naive CD4^+^ and CD8^+^ T cells in PD-1^−/−^ mice showed reduced expression of both activation- and inhibitory-associated markers (Fig. 3B), indicating an altered transcriptional state of naive T cells during early *M.tb* infection.

Further, Gene Ontology (GO) enrichment analysis reinforced these findings. Cytotoxic T cells in PD-1^−/−^ lungs were enriched for pathways related to T cell-mediated cytotoxicity, positive regulation of immune effector process, and positive regulation of cell killing (Fig. 3C), consistent with their elevated cytotoxic gene expression. Conversely, naive CD4^+^ T cells displayed enrichment in pathways involved in cell proliferation, such as leukocyte proliferation, lymphocyte proliferation, T cell proliferation (Fig. 3C), potentially reflecting compensatory expansion in response to dysregulated activation.

Transcriptional heterogeneity among pulmonary T cells revealed distinct activation and differentiation states. To further explore the developmental relationships within this immune landscape, pseudo-temporal trajectory analysis of T cells was performed, which identified three sequential states (Fig. 3D). State 1, representing the inferred developmental origin, was dominated by naive CD4^+^ T and naive CD8^+^ T cells (Fig. 3D and E). Cells in State 2, primarily cytotoxic T and effector T cells, and State 3, encompassing Tregs, Th17, and Tfh cells, possessed relatively higher pseudo-temporal values, suggesting more advanced differentiation-associated transcriptional states (Fig. 3D and E). Strikingly, PD-1^−/−^ mice lungs exhibited a marked reduction of cytotoxic T cells but an expansion of effector T cells within State 2 (Fig. 3E). This altered distribution of cytotoxic T cells in PD-1^−/−^ mice lungs was accompanied by an increased representation of immunomodulatory subsets within State 3 (Tregs, Th17, and Tfh cells), suggesting a potential role for PD-1 in maintaining the balance between effector and regulatory T-cell states. Trajectory-associated gene expressions further revealed distinct transcriptional features across states: cells in State 2 upregulated cytotoxic effectors (e.g., *Gzma*, *Gzmb*, *Nkg7*, *Cd8a*, *Cd28*, *Lck,* etc.), while cells in State 3 expressed genes associated with helper and regulatory functions (e.g. *Cxcr6*, *Gata3*, *Il1rl1*, *Cd69*, *Fos*, etc.) (Fig. 3F). These data collectively suggest that PD-1 deficiency is associated with altered pulmonary T-cell developmental trajectories and transcriptional programs, favoring regulatory and helper T-cell-associated states while reducing the representation of cytotoxic T cells during the early phase of *M.tb* infection.

### PD-1 deficiency reprograms transcriptional dynamics of pulmonary B lymphocytes during early *M.tb* exposure

**Fig 4:**
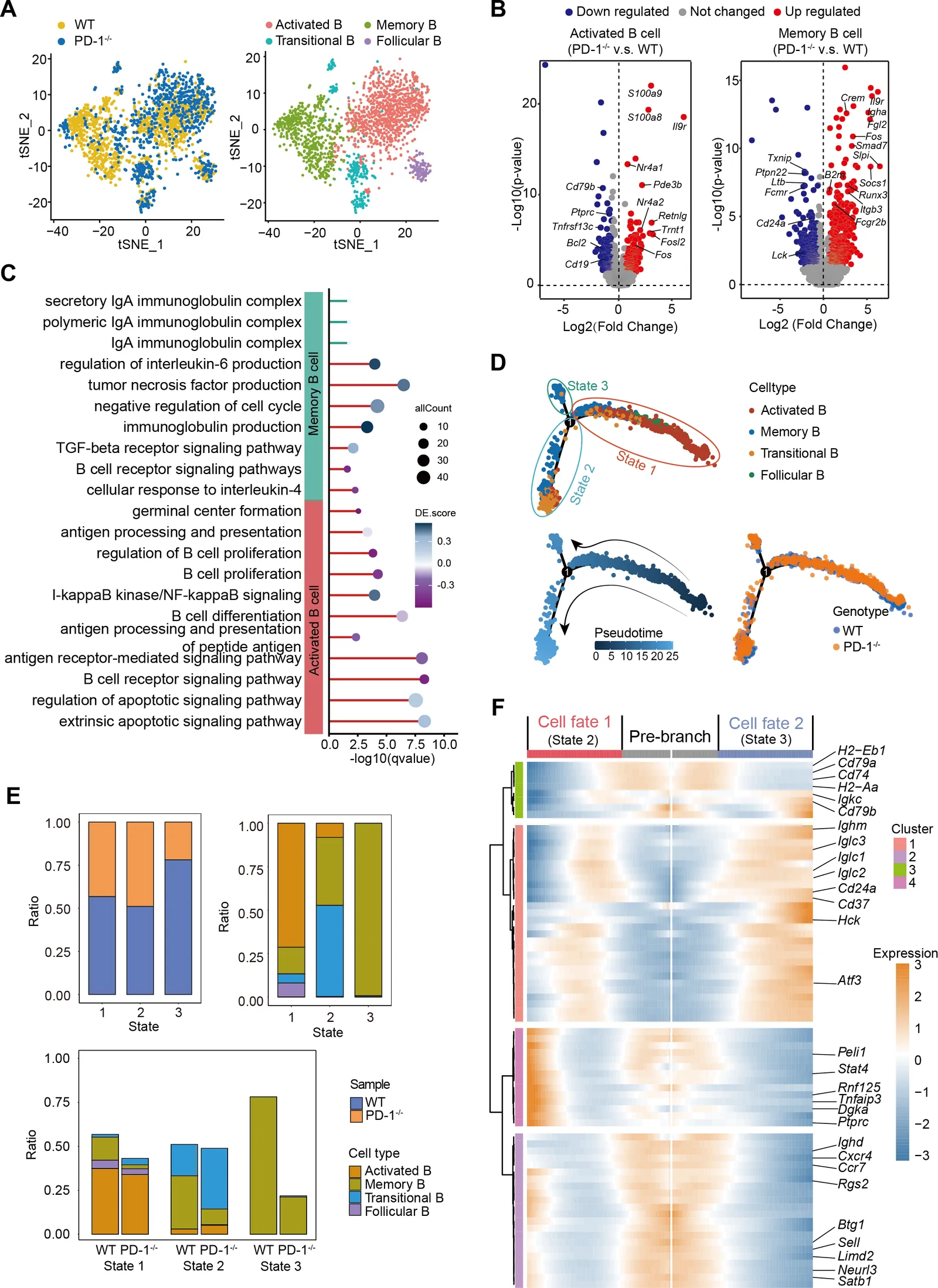
PD-1 deficiency remodels the transcriptional profiles and differentiation dynamics of pulmonary B lymphocytes during early *M.tb* infection. B cell subsets were extracted from pooled lung cells of three mice per group. (**A**) t-SNE plot of lung-derived B cells from WT and PD-1^−/−^ mice, with color-coded by genotype (left panel) and annotated B cell subsets (Right panel). (**B**) Differentially expressed genes between WT and PD-1^−/−^ groups among indicated B cell clusters. (**C**) Enriched pathways of differential expression genes across different B cell subclusters between WT and PD-1^−/−^ groups. The color scale represents the absolute value of differential expression (DE) score. Positive values indicate that >50% of genes in the pathway are upregulated, while negative values indicate predominant downregulation. (**D**) Pseudotime trajectory analysis of B cell differentiation constructed by Monocle. Top: cell distribution along the developmental trajectory, annotated by B cell subsets and cell states. Bottom left: Cells ordered by pseudotime. Bottom right: cells grouped by sample origin (WT and PD-1^−/−^). (**E**) Proportional distribution of WT and PD-1^−/−^ cells, B subsets, B subsets from different groups in different states. (**F**) Hierarchical clustering heatmap of differently expressed genes between WT and PD-1^−/−^ B cells along with the pseudotime trajectory. The color scale represents relative gene expression levels.

PD-1-deficiency-driven heterogeneities in cellular subsets and transcriptional programs were also observed in pulmonary B lymphocytes. The marked reduction of memory B cells in lungs of PD-1^−/−^ mice highlights PD-1 as a critical regulator of memory B cell lineage commitment in pulmonary anti-mycobacterial response (Fig. 2C, I and 4A). Moreover, PD-1^−/−^ memory B cells exhibited a compensatory upregulation of negative regulatory genes (e.g., *Socs1*, *Fgl2*, *Smad7,* and *Crem*) (Fig. 4B), indicating PD-1 deficiency disrupts the homeostatic regulatory mechanisms of memory B cells. While the abundance of activated B cells in PD-1^−/−^ mouse lungs remained unchanged, their transcriptional landscape undergoes a pronounced shift toward a pro-inflammatory phenotype, characterized by elevated expression of alarmins (*S100a8*, *S100a9*, *Retnlg*) and AP-1 (*Fos, Fosl2*) genes (Fig. 4B). This inflammatory skewing coincided with impaired B cell receptor (BCR) signaling, as evidenced by dysregulation of key molecules (e.g., *Cd19*, *Ptprc,* and *Cd79b* in PD-1^−/−^ activated B cells, *Lck,* and *Cd24a* in PD-1^−/−^ memory B cells) (Fig. 4B). These findings highlight PD-1’s function in restraining excessive inflammation while maintaining the precision of antigen-specific B cell responses.

This oppositional effect was further confirmed by enrichment analysis on transcriptomic change. PD-1^−/−^ activated B cells exhibited augmented apoptotic and NF-κB signaling, alongside suppression of B cell activation signals such as germinal center formation, B cell receptor signaling pathway, B cell differentiation, and B cell proliferation (Fig. 4C). Similarly, memory B cells from PD-1-deficient lungs displayed enhanced proinflammatory signaling pathways (e.g., IL-6 production and TNF production), accompanied by suppressed BCR signaling pathway and cellular responses to IL-4 pathway (Fig. 4C). The upregulation of suppressive axes (e.g., TGF-β receptor signaling pathway) following PD-1 deficiency further underscores its non-redundant role in homeostatic maintenance. Collectively, these results revealed the multifaceted role of PD-1 in modulating distinct B cell subsets during early *M.tb* exposure and highlighted its capacity to orchestrate an effective protective immune response.

Developmental trajectory reconstruction resolved three putative maturation states of pulmonary B lymphocytes in the early stage of *M.tb* infection. State 1, positioned at the trajectory origin (Pre-branch) (Fig. 4D and F), was enriched for activated B cells and memory B cells. State 2 (Cell fate 1 in Fig. 4F) comprised transitional B cells and memory B cells (Fig. 4E). State 3 (Cell fate 2 in Fig. 4F) represented a terminal memory-associated state exhibiting co-expression of memory markers (*Ighm*, *Iglc1/2/3*, *Cd24a*) (Fig. 4F). Across all three states, memory B cells were predominantly WT-derived (Fig. 4E). Although PD-1^−/−^ B cells displayed a trajectory architecture similar to that of WT B cells, an altered endpoint distribution marked by a striking reduction of State 3 cells (memory B cells) was observed in PD-1^−/−^ mice (Fig. 4E), suggesting that PD-1 deficiency may be associated with impaired progression toward memory B cell-linked states in mice lungs. In summary, our findings indicate a regulatory role of PD-1 in maintaining both pulmonary B-cell functional coordination and developmental programs during early *M.tb* infection.

### PD-1 coordinates pulmonary lymphocyte states through predicted transcriptional regulatory networks

**Fig 5:**
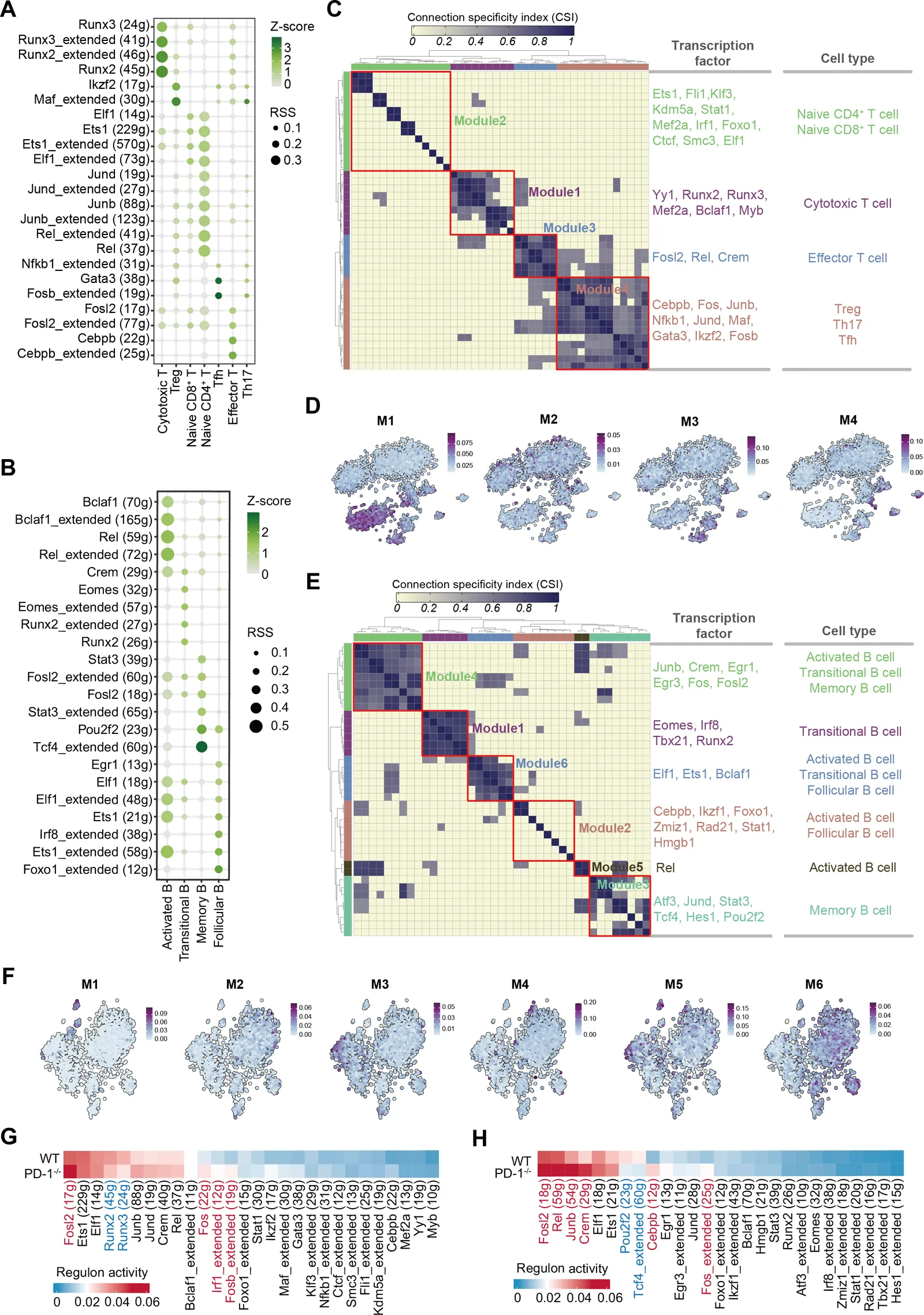
PD-1 deficiency reprograms transcriptional signatures through distinct gene regulatory networks in T and B lymphocytes. Lymphocyte subsets were extracted from pooled scRNA-seq data of three mice per group for downstream analysis. (**A and B**) The RSS of specific transcriptional regulators associated with distinct T (**A**) and B (**B**) cell subpopulations. (**C**) Regulatory network modules identified in T cells based on the regulator connection specificity index (CSI) matrix, along with representative transcriptional factors and associated cell types. (**D**) Visualization of average regulon module activity scores projected onto the *t*-SNE embedding of T cells. The color scale represents average regulon activity scores. (**E**) Regulatory modules identified in B cells based on regulon CSI matrix, along with representative transcriptional factors and associated B cell subtypes. (**F**) Average regulon module activity scores projected onto *t*-SNE map of B cells. The color scale represents average regulon activity scores. (**G and H**) The regulon activity scores (RAS) of individual transcriptional regulators in T cells (**G**) and B cells (**H**) from WT and PD-1^−/−^ mice. The color scale represents relative regulon activities.

To explore the potential upstream mechanisms associated with PD-1-driven transcriptional reprogramming of pulmonary lymphocytes under *M.tb* infection, we constructed the transcriptional regulatory networks using single-cell regulatory network inference and clustering (SCENIC) analysis (33). By calculating the regulon specificity score (RSS) (34), we identified predicted lineage-associated transcription factors (TFs) across distinct lymphocyte subsets (Fig. 5A and B). In pulmonary T cells, *Runx2* and *Runx3* were identified as the most specific regulons associated with cytotoxic T cells, *Maf* and *Ikzf2* as the top regulons associated with Tregs, while *Cebpb* and *Fosl2* specified effector T cell identity (Fig. 5A). For B cell populations, *Bclaf1*, *Rel*, Ets1, and *Elf1* were associated with activated B cells, whereas *Tcf4*, *Pou2f2*, *Fosl2,* and *Stat3* were among the top regulons enriched in memory B cells (Fig. 5B). PD-1 deficiency was associated with elevated RSS scores for Treg (*Maf*, *Ikzf2*) and Tfh (*Gata3*, *Fosb*) while suppressing key regulators of cytotoxic T cells (*Runx2/3*) (Fig. S2A). Similarly, in B cells, PD-1 deficiency increased RSS scores for transitional B cell-associated regulators (*Eomes*) while decreasing those associated with memory B cell identity (*Tcf4*, *Pou2f2*) (Fig. S3A). These transcriptional changes were consistent with the altered T-cell and B-cell developmental trajectories inferred from pseudotime analysis, suggesting that PD-1 may contribute to the maintenance of pulmonary lymphocyte state stability during early *M.tb* infection.

To systematically dissect the combinatorial patterns across lymphocytic subsets, we calculated the connection specificity index (CSI) and identified distinct regulon modules with cell-type-specific activation profiles (35, 36). In pulmonary T cells, the transcriptional programs were organized into four major modules, each defined by several representative regulators and cell types which were identified by mapping the average module activity score onto tSNE map (Fig. 5C and D). Module 1, defined by *Runx2* and *Runx3*, was associated with cytotoxic T cell differentiation programs. Module 2, enriched in regulators associated with naive T cells, such as *Ets1* and *Elf1*, represented early T cell developmental states. Module 3, characterized by *Fosl2*, *Rel,* and *Crem*, was associated with effector T-cell activation programs. Module 4 comprised a mixture of helper T cell-associated regulators, such as *Maf*, *Ikzf2*, *Gata3,* and *Fosb*, which was enriched for helper T cell-associated programs, including Th17 and Tfh polarization. The combinatorial pattern analysis for B cell subpopulations identified 6 major modules (Fig. 5E and F). Module 1, enriched for *Eomes, Irf8, Tbx21,* and *Runx2*, was associated with transitional B cell maturation. Module 2 contained several key regulators of B cell development and germinal center formation, such as *Cebpb*, *Ikzf1,* and *Foxo1*, which are enriched in activated B cells and follicular B cells. The regulons in Module 3 were associated with memory B cell-related transcriptional programs, such as *Tcf4*, *Pou2f2,* and *Stat3*. Module 4, dominated by activator protein-1 (AP-1) (*Junb*, *Fos*, *Fosb*) and early growth response gene (Egr) family (*Egr1*, *Egr3*), regulated cellular proliferation, transformation, and apoptosis. Regulon in Module 5, *Rel*, was linked to BCR signaling activity in activated B cells. Module 6, encompassing *Ets1*, *Elf1*, and *Bclaf1*, functioned as a differentiation restraint module, maintaining activated B cells, transitional B cells, and follicular B cell stability.

Next, comparative analysis of the regulon activity scores (RAS) revealed PD-1 deficiency was associated with AP-1 activity in both pulmonary B and T cells, as indicated by elevated *Fosl2*, *Junb*, *Jund*, *Fos,* and *Fosb* regulon activity (Fig. 5G and H; Fig. S2B and D, S3B and D), linking PD-1 deficiency to excessive inflammatory signaling. Concurrently, reduced activity of *Runx2* and *Runx3* regulons in PD-1 deficient pulmonary T cells was observed (Fig. 5G; Fig. S2C and D), consistent with altered T lymphocyte differentiation- and development-associated transcriptional programs in mice lungs. In PD-1^−/−^ Tregs, heightened *Ikzf2* and *Foxo1* regulon activity (Fig. S2C), which is indispensable for maintaining the functions and stability of Tregs (37, 38), may reflect compensatory regulatory function. Similarly, PD-1 deficiency was associated with increased AP-1- and Egr-related regulon activity (*Fosl2*, *Junb*, *Junb,* and *Fos*) and decreased activity of memory B cell-associated regulons (*Tcf4*, *Pou2f2*) during early stage of *M.tb* exposure (Fig. 5H; Fig. S3C). Collectively, these findings suggest that PD-1 deficiency is accompanied by broad alterations in predicted transcriptional regulatory networks that may contribute to changes in lymphocyte maturation and functional states during early *M.tb* infection. In addition, these transcriptional perturbations may influence cell-cell communication patterns, potentially affecting coordinated immune responses.

### PD-1 deficiency disrupts early B-T cell communication during *M.tb* infection

**Fig 6:**
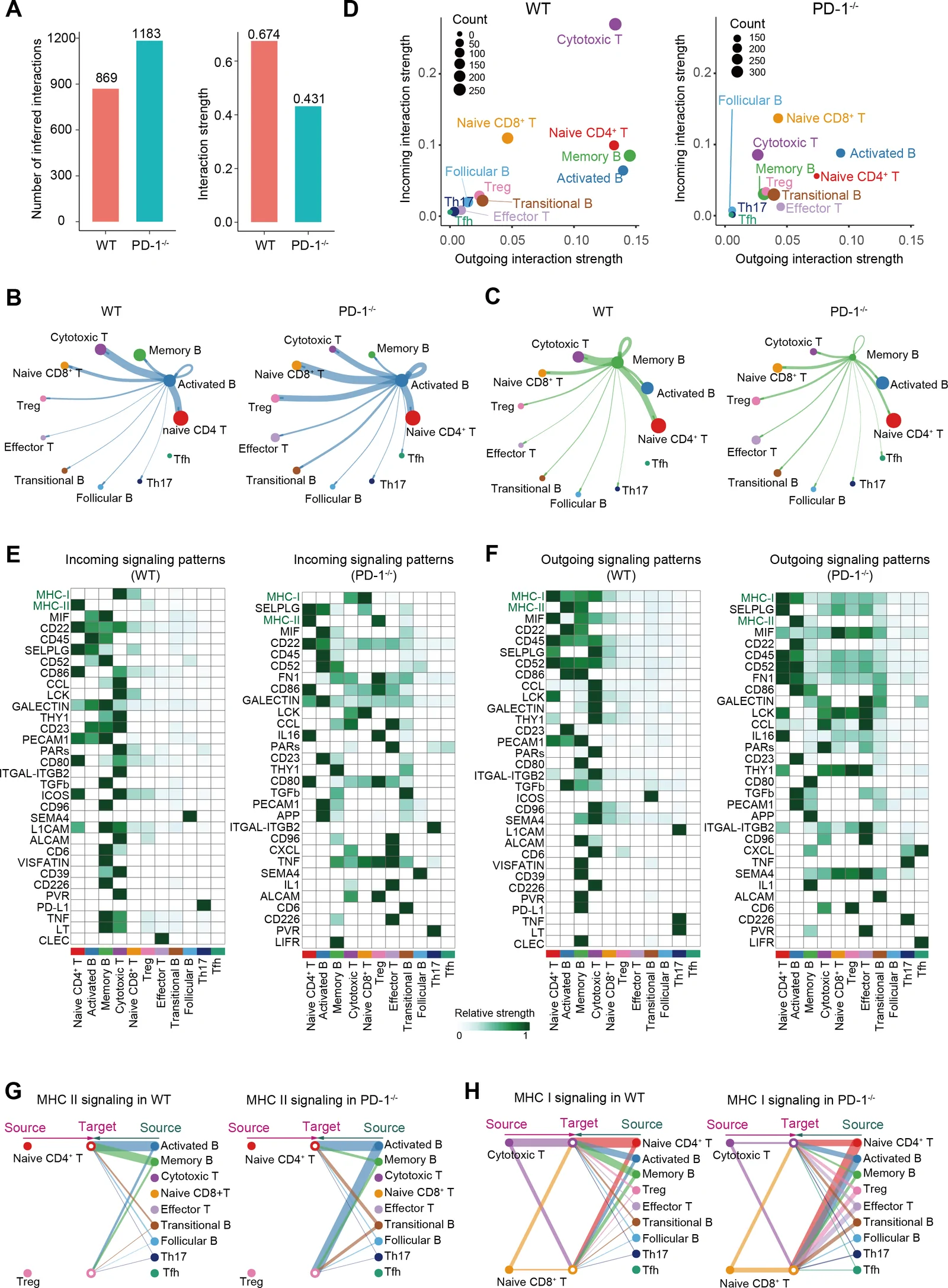
PD-1 deficiency disrupts the balance of B-T cell intercellular communication during early *M.tb* infection. Lymphocyte subsets were extracted from pooled scRNA-seq data of three mice per group for downstream analysis. (**A**) Overall comparison of the inferred interaction numbers and strengths between WT and PD-1^−/−^ groups. (**B and C**) Quantitative comparison of outgoing signaling interactions from activated B cells (**B**) and memory B cells (**C**) to other immune cell types in WT and PD-1^−/−^ groups. (**D**) The global interaction strength profiles of all detected cell types. Dot color indicates specific cell identity, and dot size reflects the total interaction numbers associated with each cell type. (**E and F**) Relative contributions of individual cell populations as signal receivers (**E**) and signal senders (**F**) across major signaling pathways, comparing WT and PD-1^−/−^ conditions. The color scale represents relative strength of incoming (**E**) or outgoing (**F**) signaling. (**G**) The inferred MHC class II signaling networks while naive CD4^+^ T cells and Tregs as the signal receivers. (**H**) The inferred MHC class I signaling networks while cytotoxic T cells and naive CD8^+^ T cells as the signal receivers.

To assess whether PD-1-deficiency-mediated transcriptional alterations impact early communication between pulmonary B and T lymphocytes under *M.tb* infection, we analyzed the gene expression-based cell-cell interaction patterns within *M.tb*-infected WT and PD-1^−/−^ mice lungs. Notably, PD-1^−/−^ mice lungs exhibited an increase in total interaction numbers but a decrease in overall interaction strength (Fig. 6A). Specifically, naive CD8^+^ T cells and Tregs in PD-1^−/−^ lungs received stronger incoming signals from activated B cells, whereas cytotoxic T cells received fewer signals from both activated B and memory B cells (Fig. 6B and C; Fig. S4A). In WT lungs, cytotoxic T cells, naive CD4^+^ T cells, memory B cells, and activated B cells served as the predominant sources of strong outgoing signals. However, these signals were notably attenuated in PD-1^−/−^ mice, particularly those originating from cytotoxic T cells and memory B cells (Fig. 6D). Conversely, signaling from Tregs, transitional B cells, and effector T cells increased in PD-1^−/−^ lungs (Fig. 6D), indicating a rewiring of potential early lymphocyte interaction networks. These findings suggest that PD-1 deficiency is associated with altered patterns of lymphocyte interactions during the early phase of *M.tb* infection, potentially reshaping the pulmonary lymphocytic environment.

We next compared the global information flow across major signaling pathways to determine how PD-1 loss affects the overall communication probability (Fig. S4B and C). Pathways related to co-stimulatory and antigen presentation, such as ICOS, CD80, CD86, CD6, MHC-I, MHC-II, CD23, LCK, CD45, and CD226, were more active in WT lungs (Fig. S4B). In contrast, pro-inflammatory signaling pathways like TNF, IL1, and IL16, were more active in PD-1^−/−^ mice lungs, suggesting a skewing towards inflammatory responses in the absence of PD-1 (Fig. S4B). Despite these shifts, MHC-I and MHC-II signaling pathways remained dominant in both WT and PD-1^−/−^ lungs (Fig. S4C), reflecting that antigen processing and presentation continued to be central to immune responses during early *M.tb* exposure.

A detailed analysis of the MHC-II signaling pathway revealed notable alterations in both signal reception and transmission in PD-1^−/−^ lungs. PD-1^−/−^ Tregs exhibited enhanced integration of MHC-II signals compared with WT Tregs (Fig. 6E), while PD-1^−/−^ transitional B cells exhibited increased outgoing MHC-II signals at the expense of memory B cells (Fig. 6F). When examining MHC-II signaling networks involving naive CD 4^+^ T cells and Tregs as receivers, we found that PD-1 deficiency led to reduced signals from memory B cells but increased from transitional B cells. Meanwhile, PD-1^−/−^ Treg received heightened input from both activated and transitional B cells (Fig. 6G). Corresponding ligand-receptor pair analysis (e.g., *H2-aa-Cd4*, *H2-ab1-Cd4*, *H2-dma-Cd4*, etc.) confirmed this pattern, showing increased signal probability from PD-1^−/−^ activated and transitional B cells to Tregs, with a concurrent reduction in signals to naive CD4^+^ T cells (Fig. S4D). These findings suggest that, in the absence of PD-1, predicted communication patterns become increasingly enriched toward Tregs, accompanied by reduced interactions involving conventional CD4^+^ T cells.

In the context of MHC-I signaling, cytotoxic T cells and naive CD8^+^ T cells were the primary signal recipients (Fig. 6E). PD-1^−/−^ cytotoxic T cells received stronger MHC-I signals from Tregs, effector T cells, and transitional B cells, but weaker MHC-I signals from cytotoxic T, naive CD4^+^ T, activated B, and memory B cells (Fig. 6H). Correspondingly, ligand-receptor pairs analysis revealed a reduction of MHC-I signals interactions involving naive CD4^+^, activated B, and memory B cells with cytotoxic T cells, while naive CD8^+^ T cells in PD-1^−/−^ lungs exhibited broader engagement with multiple cell types (Fig. S4E. Interestingly, PD-1 deficiency led to a loss of *H2-q6-Cd8a* and *H2-q6-Cd8b1* expression-based interactions but gained novel predicted interactions, including *H2-t23-Cd8a*, *H2-t23-Cd8b1*, *H2-t23-(Klrd1 + Klrc1*), and *H2-t23-(Klrd1 + Klrc2*) between B cells and cytotoxic T cells (Fig. S4E). Together, these findings revealed that PD-1 deficiency leads to widespread transcriptional reprogramming of ligand-receptor networks among pulmonary lymphocytes during *M.tb* infection. These communication defects may have far-reaching consequences on the orchestration of adaptive immune responses and the development of long-term immunity during *tuberculosis*.

## DISCUSSION

**Fig 7:**
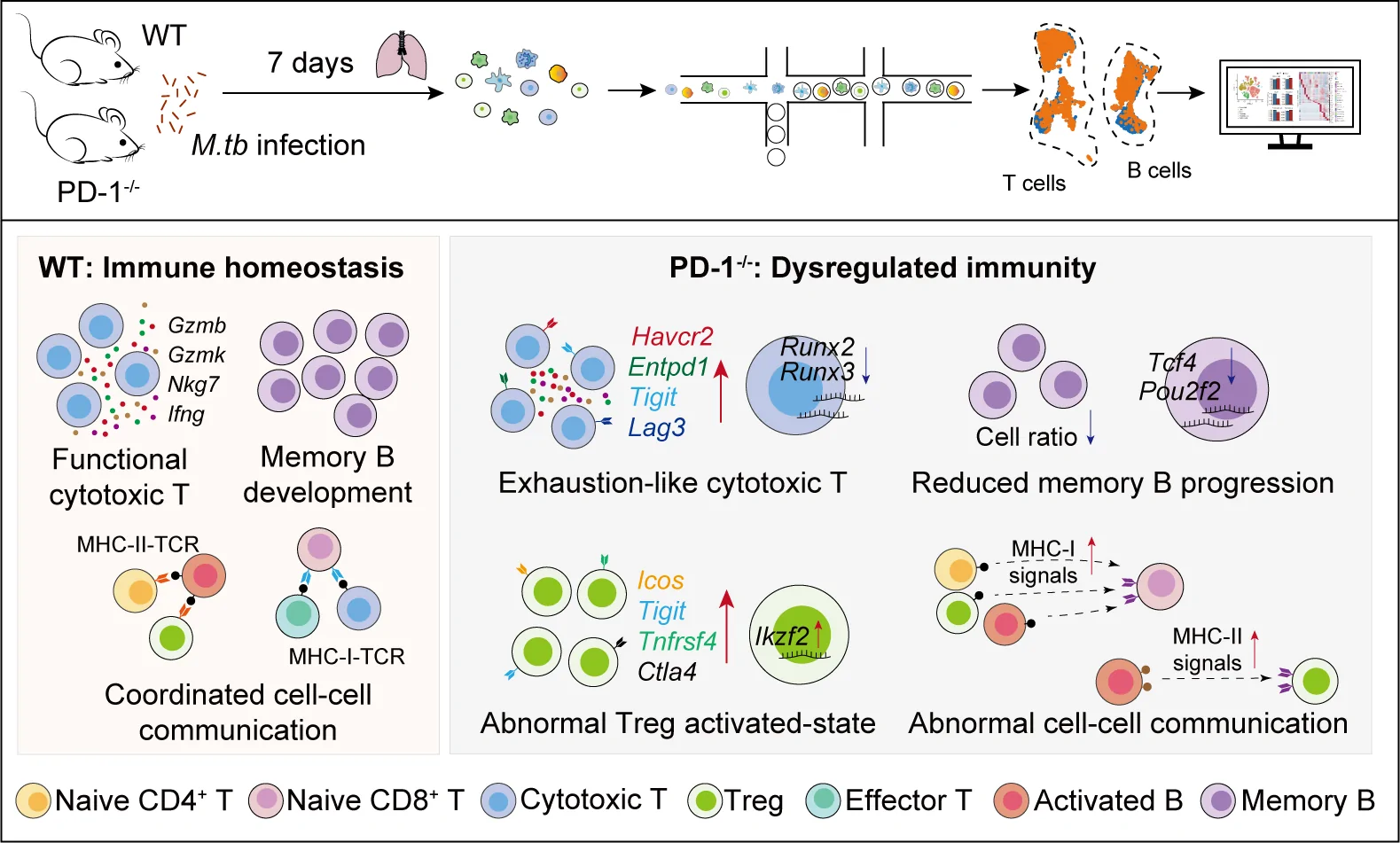
Schematic summary of PD-1 in regulating pulmonary lymphocyte responses during early *M.tb* infection. PD-1 deficiency induces transcriptional reprogramming characterized by increased Ikzf2 expression and reduced Runx2, Runx3, Tcf4, and Pou2f2 expression. These alterations promote Treg expansion, exhaustion-like differentiation of cytotoxic T cells, impaired memory B cell maturation, and disrupted B-T cell communication, resulting in immune dysregulation.

PD-1 has been extensively studied for its role in inhibiting pathogen-specific T cell responses during chronic infections and tumor-specific T cell function in cancer. However, the paradoxical effects observed during anti-PD-1 therapy, where boosting T cell responses can lead to TB, raises questions about the role of PD-1 in anti-*M*.*tb* infection (25, 27, 39, 40). Despite numerous research have been directed towards understanding PD-1’s role in chronic TB, there remains a gap in knowledge regarding how PD-1 deficiency influences the early immune responses of lymphocytes following the initial stage of *M.tb* exposure. Our research reveal that PD-1 serves as a critical orchestrator of early lymphocyte fate determination during *M.tb* infection, extending its conventional role in T cell exhaustion in cancer and chronic infections (26). In this study, we characterized the transcriptional profiles, developmental states, and predicted communication networks of pulmonary B and T lymphocytes during the early stage of *M.tb* infection. Our findings revealed that genetic PD-1 deletion leads to a skewed immune response characterized by an expanded Treg proliferation, exhausted cytotoxic T cells, and delayed memory B cell development in mice lungs. These alterations are driven by a complex reprogramming of transcriptional networks, which likely impacts intercellular communication, immune cell priming, and the long-term immune fate in response to *M.tb* infection (Fig. 7).

While PD-1 is widely acknowledged for its inhibitory function in chronic settings, our results reveal that its absence during the early phase of *M.tb* infection precipitates a profound imbalance in pulmonary T cell subsets. Specifically, the loss of PD-1 leads to an expanded Treg population and exacerbates Treg activation, as evidenced by elevated co-stimulatory molecule expression. Although Tregs are essential for preventing immunopathology, excessive Treg activity may suppress protective antimycobacterial immunity and limit effective T-cell priming (41, 42). Even though it is not clear whether these Treg cells curb excessive inflammation or restrict immunity to bacteria under the condition of PD-1 deletion, Treg activation may be a marker of immunity imbalance during the initial course of TB infection. In our study, this heightened Treg activity appears to divert essential activation signals away from naive CD4^+^ T cells, thereby compromising their priming.

Cytotoxic T cells help control *M.tb* infection by directly killing infected cells, secreting cytokines, promoting granuloma formation, and establishing immune memory (14, 15, 43, 44). If cytotoxic T cells decrease or their function is impaired, the host’s immune response will be severely compromised, leading to the chronicity of *M.tb* infection, immune evasion, treatment failure, and increased susceptibility to reinfection (43, 45). Our research not only suggests the reduction of cytotoxic T cells but also reveals that these cells exhibit a paradoxical higher expression of effector molecules (*Gzma*, *Gzmb*, *Gzmk*, *Prt1*, *Nkg7*, *Ifng*) and exhausted markers (*Prdm1*, *Entpd1*, *Havcr2*, *Tigit*, *Lag3*), suggesting the emergence of an exhaustion-like phenotype. This exhausted-like phenotype also suggests early cytotoxic responses are initial but failed to mature effectively in the absence of PD-1. One possible explanation for this early exhaustion-like differentiation is that PD-1 deficiency removes a critical negative-feedback mechanism that normally restrains T-cell activation during the initial immune response. Sustained hyperactivation has been reported to accelerate terminal differentiation and trigger exhaustion-associated transcriptional programs even before chronic antigen exposure is established (46, 47). In the absence of PD-1 signaling, cytotoxic T cells may undergo excessive activation, thus accelerate terminal differentiation and trigger exhaustion-associated transcriptional programs even before chronic antigen exposure is established.

The transcriptional alterations identified in PD-1^−/−^ mice provide a mechanistic framework linking PD-1 signaling to T cell dysfunction or exhaustion-like states. Runx3 is a central regulator of cytotoxic lineage commitment, effector differentiation, and memory formation, whereas Runx2 contributes to T cell activation and survival (48–50). Reduced expression of *Runx2* and *Runx3* may therefore compromise the development and maintenance of functional cytotoxic T cells. In contrast, increased expression of *Ikzf2* (*Helios*), a transcription factor associated with Treg stability and suppressive activity (37, 38), may reinforce regulatory programs at the expense of protective effector responses. Together, these transcriptional changes likely contribute to the expansion of activated Tregs and the premature exhaustion-like differentiation of cytotoxic T cells observed in PD-1^−/−^ mice, ultimately weakening adaptive immune responses against *M.tb*. Similarly, within the B-cell compartment, *Tcf4* and *Pou2f2* are critical regulators of B-cell development, germinal center responses, and antigen-driven differentiation (51–53). Downregulation of these transcription factors may impair memory B-cell generation and maturation, reducing the capacity to establish durable humoral immunity. Because effective protection against tuberculosis requires coordinated interactions between cellular and humoral immune responses, disruption of these transcriptional programs may collectively weaken immune containment of *M.tb* and promote disease progression. Therefore, the altered expression of *Runx2*, *Runx3*, *Ikzf2*, *Tcf4*, and *Pou2f2* likely represents a central molecular framework through which PD-1 deficiency reshapes lymphocyte fate decisions and compromises immune homeostasis during early TB.

Recent studies identified that PD-1-deficient humans and mice exhibit fewer memory B cells and impaired antibody responses, which suggests that ablation of PD-1 signaling contributes to B cell dysregulation (54). Our analysis of the pulmonary B cell compartment further reveals that PD-1 deficiency impairs critical processes involved in B cell maturation, especially memory B cell formation, following early *M.tb* exposure. Memory B cells are essential for generating rapid and robust antibody responses upon re-exposure to pathogens, playing a critical role in long-term humoral immunity (10, 55–57). In PD-1^−/−^ mice, we observed a reduced frequency of memory B cells and reduced representation of memory B-associated developmental states. Importantly, this phenotype was accompanied by decreased activity of predicted regulators linked to memory B-cell differentiation, including *Tcf4* and *Pou2f2*. Another finding of B cell subpopulations was the lower expression of MHC molecules on activated B cells, accompanied by the downregulation of pathways associated with antigen presentation in PD-1^−/−^ mice compared with the WT group. Because activated B cells serve as APCs and engage in cognate interactions with T cells, thereby facilitating affinity maturation and subsequent differentiation into memory B cells or antibody-secreting plasma cells (20, 58, 59). In PD-1^−/−^ mice, the impaired antigen presentation capacity of activated B cells likely disrupts their ability to effectively engage with T cells, potentially compromising T cell activation and the subsequent feedback signals that drive B cell differentiation. Furthermore, we observed elevated expression of transcription factors such as *Fosl2*, *Junb*, *Jund*, *Rel*, and *Crem* in PD-1^−/−^-activated B cells, which are associated with cell proliferation, survival, and stress responses (60, 61). While these factors may enhance B cell activation, their overexpression could also skew B cell differentiation, leading to an imbalance in the generation of effector and memory B cell populations. As we have indicated, genetic PD-1 deletion has indeed impaired the development and maturation of memory B cells.

The alterations in the pulmonary lymphocytic ecosystem of PD-1^−/−^ mice following early *M.tb* exposure also included imbalanced inferred intercellular communication. It is well known that B cells act as APCs to activate T cells, while T cells provide critical signals such as CD40L and cytokines (e.g., IL-21) that drive B cell differentiation (21, 22, 62). In PD-1^−/−^ mice, the delayed development of memory B cells and the altered transcriptional profiles of both B and T cell subsets in lungs suggest a breakdown in this essential crosstalk. The paradox of increased interaction numbers but diminished interaction strength of cellular communication between pulmonary lymphocytes in PD-1^−/−^ mice suggests a loss of signal specificity, wherein aberrant activation of transitional B cells and Tregs may override coordinated adaptive immunity. Notably, the enhanced MHC-II signaling to Tregs and weakened communication between memory B cells and naive CD4^+^ T cells imply a skewed immune landscape favoring regulatory over effector responses. This imbalance could permit *M.tb* persistence by dampening cytotoxic T cell priming and delaying memory B cell maturation, both essential for pathogen containment. Furthermore, the shift toward pro-inflammatory (TNF, IL1) and tissue-remodeling (APP, FN1) pathways in pulmonary lymphocytes of PD-1^−/−^ mice may reflect compensatory mechanisms attempting to counter defective adaptive immunity, inadvertently promoting chronic inflammation or fibrosis. These findings align with clinical observations of immune-related adverse events during anti-PD-1 therapy, where unchecked inflammation coexists with impaired pathogen control (63, 64). Our study thus positions PD-1 not merely as an exhaustion marker but as a linchpin in early immune coordination, whose ablation creates a dysfunctional ecosystem permissive to pathogen evasion. Future studies should explore whether timed PD-1 modulation, rather than complete ablation, could preserve beneficial interactions while mitigating pathological inflammation in *tuberculosis*.

From a translational perspective, our findings provide important insights into the potential opportunities and challenges of targeting the PD-1 pathway in TB. Although PD-1 blockade may theoretically enhance antimicrobial immunity by increasing lymphocyte activation, our data suggests that complete disruption of PD-1 signaling results in substantial immune dysregulation rather than improved immune protection. The expansion of activated Tregs, exhaustion-like differentiation of cytotoxic T cells, impaired memory B-cell maturation, and altered lymphocyte communication observed in PD-1-deficient mice collectively indicate that excessive immune activation does not necessarily translate into improved protective immunity. Importantly, these findings support growing clinical and experimental evidence that PD-1 signaling serves as a critical regulator that balances protective immunity and immune-mediated pathology (65, 66). Excessive activation of inflammatory pathways may contribute to pulmonary tissue injury, granuloma disruption, and disease progression despite increased immune cell activity (25, 31, 67). Therefore, while PD-1 remains a promising target for host-directed therapy, indiscriminate or sustained PD-1 blockade may carry substantial risks. Future therapeutic strategies may require precise temporal modulation of PD-1 signaling, careful patient selection, and combination with antimicrobial treatment to enhance pathogen control while minimizing inflammatory tissue damage. Our findings may also have implications for patients receiving PD-1-targeted cancer immunotherapy. For instance, disruption of PD-1 signaling may lead to substantial immune dysregulation rather than uniformly enhanced antimicrobial protection, indicating that PD-1 may contribute to maintaining immune homeostasis. These findings provide a potential mechanistic framework for understanding why PD-1 blockade may, in certain clinical contexts, precipitate TB reactivation or exacerbate immunopathology despite augmenting immune activation (25, 27). Therefore, careful screening for latent tuberculosis and close monitoring of patients at high risk for *M.tb* infection may be warranted when administering PD-1-targeted therapies.

Overall, our findings challenge the conventional view of PD-1 as merely an inhibitory checkpoint. Instead, PD-1 appears to play an important role in coordinating lymphocyte differentiation, predicted transcriptional regulatory programs, and immune-cell communication during the early phase of *M.tb* infection. By regulating key transcriptional networks involving Runx2, Runx3, Ikzf2, Tcf4, and Pou2f2, PD-1 helps maintain the balance between protective immunity and immune homeostasis. These findings not only advance our understanding of early tuberculosis immunobiology but also provide important considerations for the development of host-directed therapies and the safe clinical application of PD-1-targeted immunotherapies.

### Limitations of the study

Our study has several limitations. Experiments were performed in BALB/c mice infected intravenously with the attenuated *M.tb* H37Ra strain, which limits extrapolation to human tuberculosis because it does not fully recapitulate the pathology or immune dynamics of virulent strains or natural respiratory infection, including organized, caseating granulomas. Flow cytometric analyses were conducted without live/dead gating, though cell viability during lung single-cell preparation consistently exceeded 80%, suggesting minimal impact on interpretation. Despite these constraints, our findings provide mechanistic insight into how PD-1 deficiency affects pulmonary T and B cell responses, and future validation using virulent strains, natural infection routes, or human samples is needed to confirm translational relevance.

## Data Availability

The data that support the findings of this study are openly available in Zenodo at (68). The single-cell RNA sequencing data re-analyzed in this study were derived from the Gene Expression Omnibus Repository (GEO). The accession number is GSE261737. The specific subset of data used in this study was extracted based on lymphocyte clusters after preliminary cell annotation. The processed data generated in this study are available in Zenodo at (68).

## SUPPLEMENTAL MATERIAL

Fig. S1 spectrum.01199-26-s0001.tif Gating strategy for flow cytometric identification of pulmonary lymphocyte subpopulations.

Fig. S2 spectrum.01199-26-s0002.tif Transcriptional regulator profiles of pulmonary T cells and their subpopulations in WT and PD-1-deficient mice during early *M.tb* infection.

Fig. S3 spectrum.01199-26-s0003.tif Transcriptional regulator profiles of pulmonary B cells and their subpopulations in WT and PD-1-deficient mice during early *M.tb* infection.

Fig. S4 spectrum.01199-26-s0004.tif Intercellular crosstalk among different lymphocyte subsets in WT and PD-1-deficient lungs during early *M.tb* infection.

Supplemental legends spectrum.01199-26-s0005.docx Descriptive legends for Fig. S1 to S4.

ASM does not own the copyrights to Supplemental Material that may be linked to, or accessed through, an article. The authors have granted ASM a non-exclusive, world-wide license to publish the Supplemental Material files. Please contact the corresponding author directly for reuse.
