# Supplementary material for: Single-cell transcriptomics reveal PD-1-loss-driven immune dysregulation of pulmonary lymphocytes during early Mycobacterium tuberculosis infection
Source: Microbiol Spectr. 2026 Jul 24;14(9):e01199-26. doi: 10.1128/spectrum.01199-26 (PMC13532292; doi:10.1128/spectrum.01199-26)
Supplement: Supplementary file 1 [file spectrum.01199-26-s0005.docx]

PD-1 deficiency remodels the transcriptomic landscape of pulmonary lymphocytes during early *Mycobacterium tuberculosis* infection

Running title: PD-1 controls pulmonary lymphocyte homeostasis in TB

(Supplementary materials)

Bing Yang^1,2,6^, Haoqi Wang^1,2^, Siqi Zhang^1,3^, Jinru Cai^2^, Gang Cao^5^, Xuan Ma^4^*****, Jinxia Dai^1,2^*****

^1^State Key Laboratory of Agricultural Microbiology, Huazhong Agricultural University, Wuhan 430070, China

^2^College of Veterinary Medicine, Huazhong Agricultural University, Wuhan 430070, China

^3^College of Life Sciences and Technology, Huazhong Agricultural University, Wuhan 430070, China

^4^College of Agriculture, Ningxia University, Yinchuan, 750021, China

^5^Faculty of Life and Health Sciences, Shenzhen University of Advanced Technology, Chinese Academy of Sciences, Shenzhen 518107, China

^6^Present address: School of Life Sciences, Ningxia University, Yinchuan 750021, China

*****Correspondence authors:

Jinxia Dai

E-mail: [jxdai@mail.hzau.edu.cn](mailto:jxdai@mail.hzau.edu.cn);

Xuan Ma

E-mail: [maxuan@nxu.edu.cn](mailto:maxuan@nxu.edu.cn).

**Supplementary figure legends**

**Fig S1. Gating strategy for flow cytometric identification of pulmonary lymphocytes subpopulations.** (A) Representative flow cytometry plots outlining the sequential gating strategy applied to lung single-cell suspensions for identification of multiple B and T lymphocyte subset, including effector CD4^+^ T cells (CD45⁺CD3⁺CD4⁺CD44^-^CD62^-^), naive CD4^+^ T cells (CD45⁺CD3⁺CD4⁺CD44^-^CD62^+^), naive CD8^+^ T cells (CD45⁺CD3⁺CD8⁺CD44^-^CD62^+^), and memory B cells (CD45⁺CD3^-^CD19⁺ CD27^+^). (B) Gating strategy for identification of Tregs, defined as CD45⁺CD3⁺CD4⁺CD25^+^FOXP3^+^ cells, in lung tissues. (C) Gating strategy for identification of cytotoxic T cells, defined as CD45⁺CD3⁺CD8⁺GZMB^+^ cells in lung single-cell suspensions. All gating thresholds were determined using single-stained controls to ensure appropriate compensation and marker specificity.

**Fig S2. The transcriptional regulator profiles of pulmonary T cells and their subpopulations in WT and PD-1-deficient mice during early *M.tb* infection.** (A) The RSS of specific regulators associated with distinct T cell subpopulations in WT and PD-1^-/-^ lungs. Dot size and color intensity correspond to the magnitude of the RSS values, reflecting the strength and specificity of each regulator’s activity across subsets. (B) Regulatory network maps representative regulons and their predicted downstream target genes within T cells. (C) The regulon activity scores (RAS) of individual T cell subsets from WT and PD-1^-/-^ groups, illustrating global differences in transcriptional regulatory activity. (D) Ridge plots showing the distribution of RAS for representative regulators across WT and PD-1^-/-^ T cells, highlighting differential regulatory dynamics.

**Fig S3. The transcriptional regulator profiles of pulmonary B cells and their subpopulations in WT and PD-1-deficient mice during early *M.tb* infection.** (A) The RSS of specific regulators associated with distinct B cell subpopulations in WT and PD-1^-/-^ lungs. Dot size and color intensity indicate the magnitude of RSS values, reflecting the strength and specificity of each regulator within different subsets. (B) Inferred regulatory networks of the representative regulons and their predictive downstream target genes in B cells. (C) The RAS of individual B cell subsets from WT and PD-1^-/-^ groups, demonstrating alterations in transcriptional regulatory activity. (D) Ridge plots displaying the distribution of RAS values for representative regulators in WT and PD-1^-/-^ B cells.

**Fig S4.** **Intercellular crosstalk among different lymphocyte subsets in WT and PD-1-deficient lungs during early *M.tb* infection.** (A) Quantitative comparison of intercellular interaction numbers and strengths between WT and PD-1^-/-^ groups, based on inferred ligand-receptor signaling networks. (B) Inferred signaling pathways enriched in either WT or PD-1^-/-^ lungs identified through their differential intercellular interactions. (C) Ranked signaling pathways according to their relative outgoing or incoming interaction strengths in WT and PD-1^-/-^ groups. (D) Predicted communication probabilities of receptor-ligand pairs within MHC class II signaling axis across B cell subtypes (including activated B cell, memory B cell and transitional B cell) and T cell subtypes (including naïve CD4^+^ T cell and Treg) in WT and PD-1^-/-^ lungs. (E) Communication probabilities of inferred receptor-ligand pairs within MHC class I signaling axis across CD8^+^ T cell subsets (including cytotoxic CD8^+^ T cells and naïve CD8^+^ T cells) and other immune cell populations across WT and PD-1^-/-^ groups.
